# Effectiveness of different exercises in improving postural balance among Parkinson's disease patients: a systematic review and network meta-analysis

**DOI:** 10.3389/fnagi.2023.1215495

**Published:** 2023-07-17

**Authors:** Di Wang, Wen J. Cui, Zhen H. Hou, Ying Gao

**Affiliations:** ^1^Department of Sports Science, Zhejiang University, Hangzhou, China; ^2^Department of Physical Education, Zhejiang International Studies University, Hangzhou, China; ^3^Department of Orthopaedics, No. 903 Hospital of PLA Joint Logistic Support Force, Hangzhou, China

**Keywords:** RCT, exercise intervention, Berg Balance Scale, Timed-Up-and-Go time, Mini-Balance Evaluation

## Abstract

**Background:**

Exercise has been reported as an effective intervention for Parkinson's disease. However, there is still debate on the what kinds of exercises prior to choosing. This study aimed to compare and rank the different exercises that effectively enhance postural balance in Parkinson's disease patients by quantifying the information gleaned from randomized controlled trials (RCTs).

**Methods:**

We conducted a comprehensive database search, including PubMed, Cochrane Library, Embase, Web of Science, and PsycINFO. The included studies were evaluated for methodological quality by the Cochrane Risk of Bias tool.

**Results:**

The RCTs were collected between the earliest available date and March 2023. Sixty RCTs were included and the total sample size used in the study was 3,537. Thirty-five studies were defined as low risk of bias, twenty-one studies as medium risk of bias, and four studies as high risk of bias. The network meta-analysis results showed that exergaming exercise can significantly improve patients' Timed-Up-and-Go time (SUCRA = 91.5%). Dance can significantly enhance patients' Berg Balance Scale (surface under the cumulative ranking curve, SUCRA = 81.3%), and rhythmical auditory exercise can significantly improve patients' Mini-Balance Evaluation Systems Test score (SUCRA = 95.6%).

**Conclusion:**

Compared with other exercises, exergaming exercise, Dance, and rhythmical auditory exercise showed superior efficacy in improving postural balance among Parkinson's disease patients.

**Systematic review registration:**

https://www.crd.york.ac.uk/prospero/, identifier: CRD42023411918.

## 1. Introduction

Parkinson's disease is a common progressive neurodegenerative disease affecting people's quality of life and physical and mental health worldwide (Ray Dorsey et al., 2018; Zhao et al., 2021). Compared to other neurological disorders, the incidence rate of Parkinson's disease is much higher, and the patient population is expected to double from 6.2 million in 2015 up to 12 million in 2040 (Dorsey and Bloem, 2018). Impaired postural balance is the main symptom of people with Parkinson's disease, which is difficult for people to carry out daily activities (Jacobs et al., 2009), lowers their balance perception, and eventually increases the risk of falling (Adkin et al., 2003). Regarding pathophysiology and symptomatic treatments, there is no complete cure for Parkinson's disease, only a way to alleviate its symptoms (Obeso et al., 2017). Fortunately, exercise intervention can significantly improve postural balance and slow down the progression of Parkinson's disease symptoms, regardless of the stage of Parkinson's disease (Kim et al., 1996; Hirsch, 2009).

Postural balance is an essential component of the body's postural control and coordination process, which is required for maintaining steadiness during static and dynamic balance (Wikstrom et al., 2005). In previous systematic reviews and meta-analyses, researchers focused on particular exercise modalities for Parkinson's disease balance performance (Ni et al., 2018; Gomes Neto et al., 2020; de Oliveira et al., 2021). While these studies provided little insight into the benefits of exercise because the limited exercise interventions were included, there were some significant drawbacks to using conventional pairwise meta-analysis to directly compare more than two types of exercise interventions (Shen et al., 2016; Liu et al., 2019; Choi et al., 2020). Tang et al. (2019) compared eight different types of exercises for patients with Parkinson's disease and concluded that Dance was an optimal and effective option for improving the functional mobility of Parkinson's disease patients (Tang et al., 2019). Similarly, Hao et al. (2022) recently evaluated ten different exercise types and they reported that Dance, Yoga, virtual reality training and resistance training were better than other exercise interventions for patients' motor function (Hao et al., 2022). However, it is crucial to find a modality of exercise to improve the postural balance of patients with Parkinson's disease. The study needed to include more exercise types to increase our understanding of how different exercise interventions affect the postural balance and postural control of people with Parkinson's disease (Kim et al., 1996; Hirsch, 2009; Tomlinson et al., 2012; Cosentino et al., 2020).

Network meta-analysis (NMA) is a recent evidence-based methodology that compares the direct and indirect effects of multiple interventions on the disease and the ranking of each intervention (Benjamin Rouse et al., 2017), which can provide a comprehensive analysis of the improvement of postural balance for Parkinson's disease patients. This study aims to evaluate the effects of different exercise interventions on postural balance for static balance, dynamic balance, and postural control for Parkinson's disease patients and to provide evidence-based recommendations for patients and clinicians.

## 2. Materials and methods

This study has been registered with the PROSPERO, under the number CRD42023411918.

### 2.1. Data sources and searches

PubMed, Embase, PsycINFO, Cochrane Central Register of Controlled Trials (CENTRAL), and Web of Science were searched from their inception date to Mar 31, 2023. The details of search terms and search procedures were shown in Supplementary Table 1.

### 2.2. Inclusion criteria

The search strategy was constructed around the PICOS tool (Hutton et al., 2015): (P) Population: people with Parkinson's disease, the mean age ≥50 years, Hoehn and Yahr stages >1; (I) Intervention: the exercises were divided into 20 types, which was determined and their definitions were summarized in Supplementary Table 2; (C) Comparator: potential comparators were control group with wait-list control and active control for patients only; (O) Outcomes: three postural balance tests for people with Parkinson's disease, Time Up and Go (TUG), Berg Balance Score (BBS) and Mini-Balance Evaluation Systems Test (Mini-BESTest) were the outcomes of this study; (S) Study type: this study included published RCTs trials.

### 2.3. Exclusion criteria

Publications conforming to the following criteria were excluded: (1) Studies with incomplete data or statistical analyses unavailable were removed; (2) Studies from non-randomized controlled trials [including reviews, comments, letters, animal studies, protocols, conference abstracts, and case reports]; (3) If articles were repeatedly published or multiple investigations were based on the same population data, the latest research or articles with comprehensive information would be included.

### 2.4. Study selection

After the exclusion and identification of eligible articles, all relevant articles were stored in an EndNote X9 reference manager. Two reviewers (WD and CWJ) independently screened the search result and retrieved full-text articles. They extracted the relevant information, including the first author, the publication year, the study area, the follow-up time, the type of exercise interventions, the total number of included participants, population age, gender ratio, and UPDRS motor subscale.

### 2.5. Data extraction

To capture data for the inclusion, a seven-item, standardized, and pre-selected data extraction form was employed, with the following headings: (1) author, (2) year of publication, (3) the stage of Parkinson's disease, (4) mean age, (5) sample size, (6) specifics of the exercise intervention, and (7) outcomes.

### 2.6. Quality evaluation

The risk of bias (ROB) for primary outcomes in RCTs was assessed using the Cochrane Handbook version 5.1.0 tool (Green and Higgins, 2008). The following seven domains were considered: (1) randomized sequence generation, (2) treatment allocation concealment, blinding of (3) participants, (4) personnel, (5) incomplete outcome data, (6) selective reporting, and (7) other sources of bias. Trials were categorized into three levels of ROB by the number of components for which high ROB potentially existed: high risk (five or more), moderate risk (three or four), and low risk (two or less) (Higgins et al., 2011). During this process, researchers independently screened the articles and finally compared the remaining articles. Any discrepancies were discussed and resolved by a third researcher until consensus and arbitration were achieved. The risk for each primary source of bias was defined as either “low risk,” “medium risk,” or “high risk” for each included trial, and a risk of bias table was created. The risk assessment for each trial was then entered independently into Review Manager (RevMan 5.3) to generate a risk of bias summary, which was reported with the meta-analysis results.

### 2.7. Data analysis

This study was used Stata software (version 17) and analysis using Frequentist framework according to the PRISMA NMA instruction manual. We first conducted a conventional pairwise meta-analysis across comparisons available for each contrast. The *I*2 statistics were employed to evaluate the heterogeneity (Melsen et al., 2014). To reveal all available effects of each exercise intervention, a network plot was generated as a simple summary description. In the network plot, nodes represent various exercise interventions and control conditions, and the lines that connect the nodes display direct head-to-head comparisons. The number of studies was proportional to the node size and the corresponding lines. Because the presence of effect sizes was referred to continuous outcomes, standard mean differences (SMD) were calculated for each comparison using relevant group means and standard deviations (SD) from individual studies (Hedges, 1984). The 95% Credibility Interval (CI) and pooled SMD were calculated as a measurement of estimated uncertainly and pooled effect sizes.

The surface under the cumulative ranking curve (SUCRA), a simple numerical statistic summarizing the cumulative ranking probability plots for each intervention, was provided as an estimated probability that was used to rate the exercise intervention (Page et al., 2016). A higher SUCRA number implies that a specific intervention is more likely to be in the top rank or extremely effective, whilst a lower score suggests that the intervention is certain to be the worst. In the consistency test, we used the “node-splitting” technique to determine whether or not the inconsistency was generated in our network (van Valkenhoef et al., 2016). If the *P*-value is higher than 0.05, the consistency was generation (Salanti et al., 2011). For detecting whether any publication bias was generated, a comparison-adjusted funnel plot was made as a concise description (Seagroatt and Stratton, 1998), such as publication bias, selective reporting, or other bias.

## 3. Results

### 3.1. Study identification

**Figure 1:**
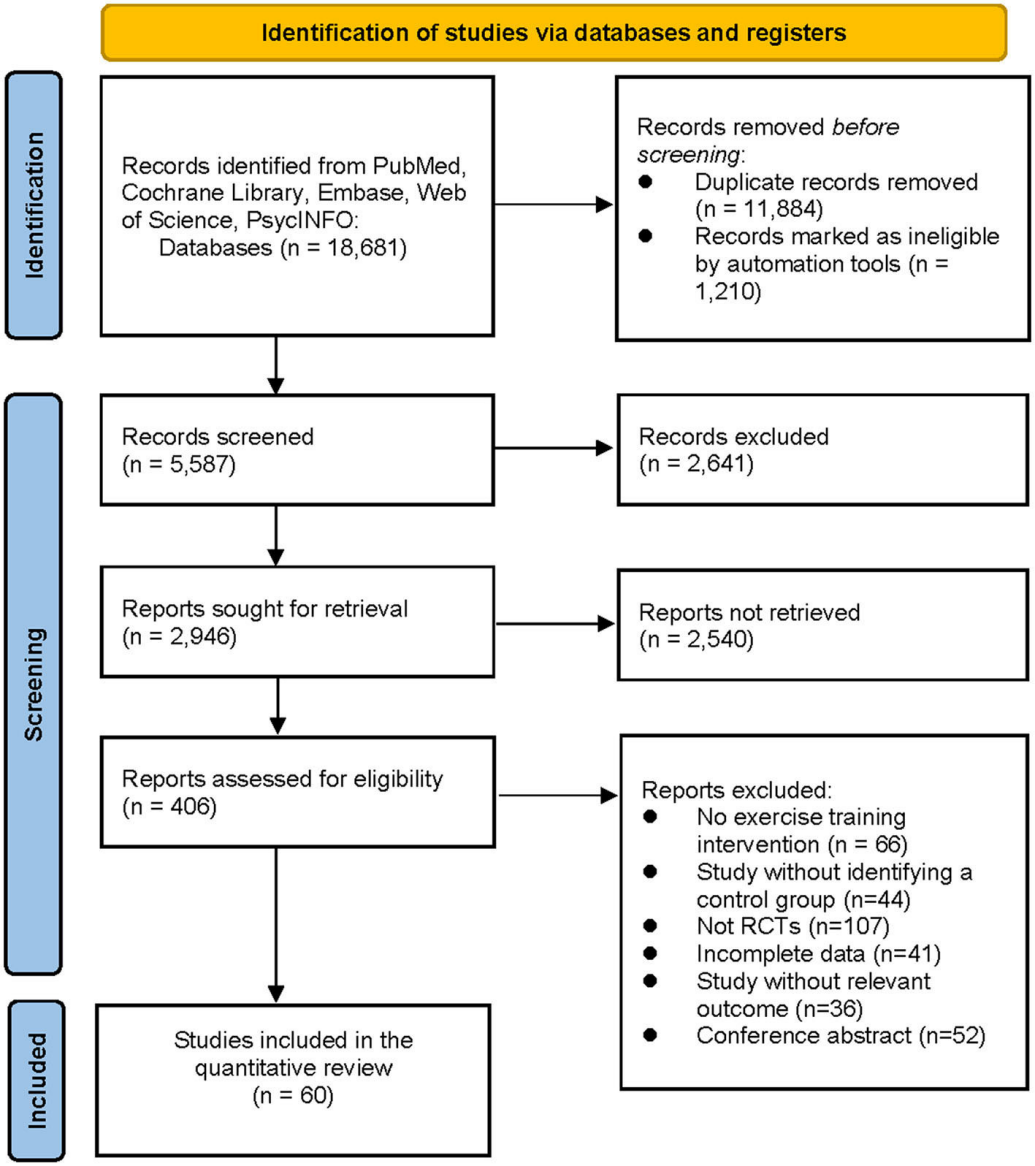
Flow diagram of the search process for studies.

A total of 18,681 articles were retrieved according to pre-established search strategies. After excluding duplicates and other reasons, the remaining 5,587 articles were read for titles and abstracts. Then 5,181 articles were excluded from irrelevant literature according to title and abstract. Investigators confirmed the 406 outcomes of interest by viewing the full texts and excluded 346 articles (for reasons including non-randomized controlled trials, incomplete data, conference papers, failure to meet the interventions, etc.). Finally, 60 articles were included in this study (Figure 1).

### 3.2. Study characteristics

This study included studies from 60 randomized controlled trials, which included 3,537 patients diagnosed with Parkinson's disease, 2,051 (57.99%) of whom were male, and 1,486 (42.01%) were female. The 60 studies were divided into 20 exercise types based on their exercise content. The exercise period ranged from 3 to 72 weeks (mean period 12.03 weeks, SD 13.23), and the frequency of exercise intervention per week ranged from 1–6 (mean frequency 2.72, SD 0. 135), and the total time of the single session ranged from 15 to 120 min (mean time 51.78 min, SD 20.86). The characteristics of the included studies were shown in Table 1 and Supplementary Table 3.

**Table 1:** Characteristics of the included studies.

**Characteristics**	**Number (%) of studies**
	**(*n* = 60)**
**Year of publication**	
2016–2023	44 (73.3)
2011–2015	14 (23.3)
2000–2010	2 (3.3)
**The mean age of participants**	
50–60	4 (6.7)
4 (60–65)	9 (15.0)
65–70	30 (50.0)
70–75	15 (25.0)
75–80	2 (3.3)
**Sample size**	
1–19	23 (38.3)
20–29	30 (50.0)
30–50	1 (1.7)
50–99	3 (5.0)
≥100	3 (5.0)
**Average disease duration (years)**	
<5	5 (8.3)
5–10	37 (61.7)
>10	14 (23.3)
Not reported	4 (6.7)
**Exercise period (weeks)**	
<8	23 (38.3)
8–12	29 (48.3)
13–24	2 (3.3)
25–48	3 (5.0)
≥49	3 (5.0)
**Disease grade (Hoehn and Yahr stage)**	
1–1.9	5 (8.3)
2–2.9	37 (61.7)
3–3.9	14 (23.3)
Not reported	4 (6.7)

### 3.3. Quality assessment

**Figure 2:**
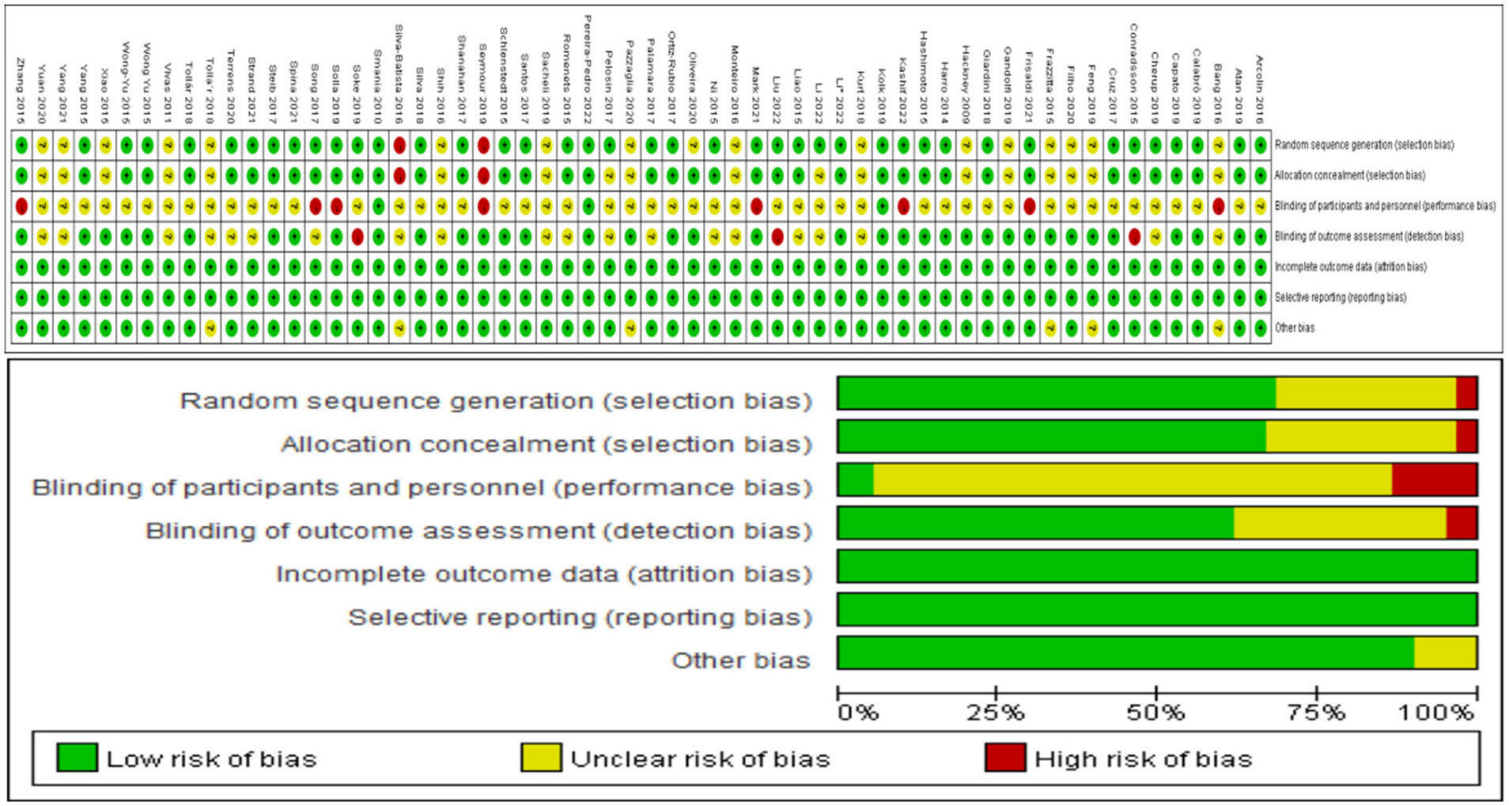
Analysis of the risk of bias in accordance with the Cochrane collaboration guideline.

The quality evaluation of RCTs revealed that the overall quality of enrolled publications was relatively high. Thirty-five studies were defined as low risk of bias, twenty-one studies as medium risk of bias, and four studies as a high risk of bias (Figure 2). Only three of these studies achieved double-blinded of subjects and measures. Because the intervention in these studies was exercise, it was difficult to achieve double-blinded to sign an informed consent form before the experiment was conducted.

### 3.4. Network meta-analysis

#### 3.4.1. Timed-Up-and-Go test

**Figure 3:**
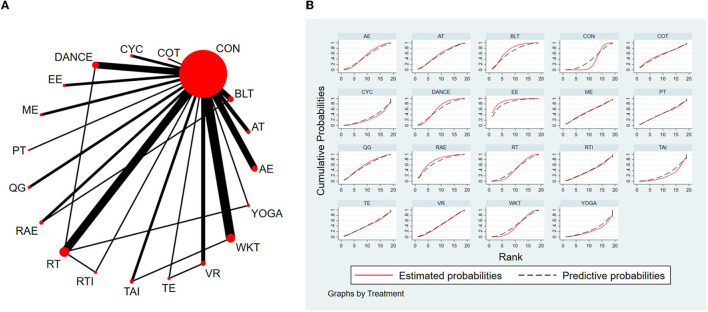
The NMA figure for TUG **(A)**. The SUCRA plot for TUG **(B)**. CON, Control Group; BLT, Balance training; AT, Aerobic training; AE, Aquatic exercise; YOGA, Yoga; WKT, Walking training; VR, Virtual reality training; TE, Traditional exercise; TAI, Tai Chi; RT, Resistance training; RTI, Resistance training with instability; RAE, Rhythmical auditory exercise; QG, Qigong; PT, Power training; ME, Multiple exercises; EE, Exergaming exercise; DANCE, Dance; CYC, Cycling training; COT, Crossover training.

**Figure 4:**
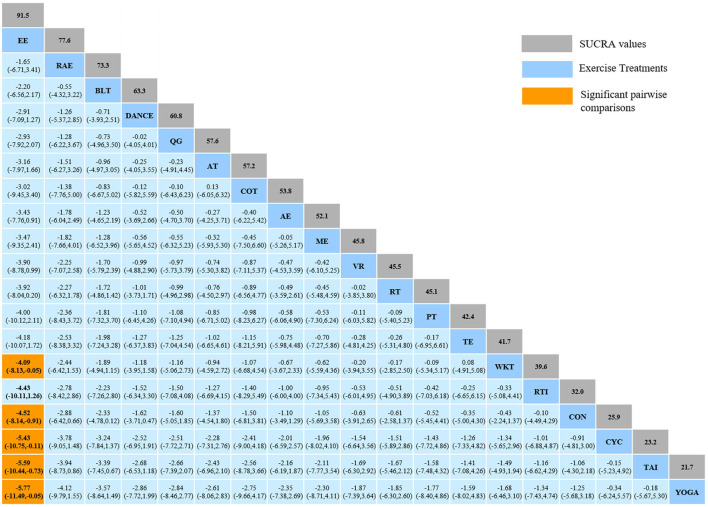
Relative effect sizes of efficacy on TUG according to network meta-analysis. CON, Control Group; BLT, Balance training; AT, Aerobic training; AE, Aquatic exercise; YOGA, Yoga; WKT, Walking training; VR, Virtual reality training; TE, Traditional exercise; TAI, Tai Chi; RT, Resistance training; RTI, Resistance training with instability; RAE, Rhythmical auditory exercise; QG, Qigong; PT, Power training; ME, Multiple exercises; EE, Exergaming exercise; DANCE, Dance; CYC, Cycling training; COT, Crossover training. Numbers in gray boxes were SUCRA values, which represented the rank of the intervention. Significant pairwise comparisons were highlighted in orange, in bold, and underlined.

The NMA for TUG were shown in Figure 3A. The probability ranking of the different exercise interventions in terms of time to TUG reduction was ranked first by exergaming exercise in the SUCRA which was shown in Figure 3B. The results of the Network meta-analysis showed that exergaming exercise (SUCRA = 91.5%), rhythmical auditory exercise (SUCRA = 77.6%), balance training (SUCRA = 73.3%), Dance (SUCRA = 63.3%), Qigong (SUCRA = 60.8%), aerobic training (SUCRA = 57.6%), crossover training (SUCRA = 57.2%), aquatic exercise (SUCRA = 53.8%), multiple exercises (SUCRA = 52.1%), virtual reality training (SUCRA = 45.8%), resistance training (SUCRA = 45.5%), power training (SUCRA = 45.1%), traditional exercise (SUCRA = 42.4%), walking training (SUCRA = 41.7%), and resistance with instability training (SUCRA = 39.6%) were higher than the control group (SUCRA = 32%) in decreasing TUG time. The exergaming exercise (EE) was significantly superior to the control group (CON) and underlying estimates of effect were presented (MD = −4.52, CI = −8.14, −0.91). Additionally, the exergaming exercise (EE) was significantly better than exercise intervention [YOGA (SMD = −5.77, CI = −11.49, −0.05), TAI (SMD = −5.59, CI = −10.44, −0.73), CYC (SMD = −5.43, CI = −10.75, −0.11), WKT (SMD = −4.09, CI = −8.13, −0.05)]. The relative effect sizes of efficacy on TUG were shown in Figure 4.

For the consistency test, the node-splitting analysis showed that all *p*-values for indirect and direct comparisons were higher than 0.05, indicating a good convergence of the model and a better-iterated effect of enrolled studies. Details were shown in Supplementary Table 4.

#### 3.4.2. Berge balance scale

**Figure 5:**
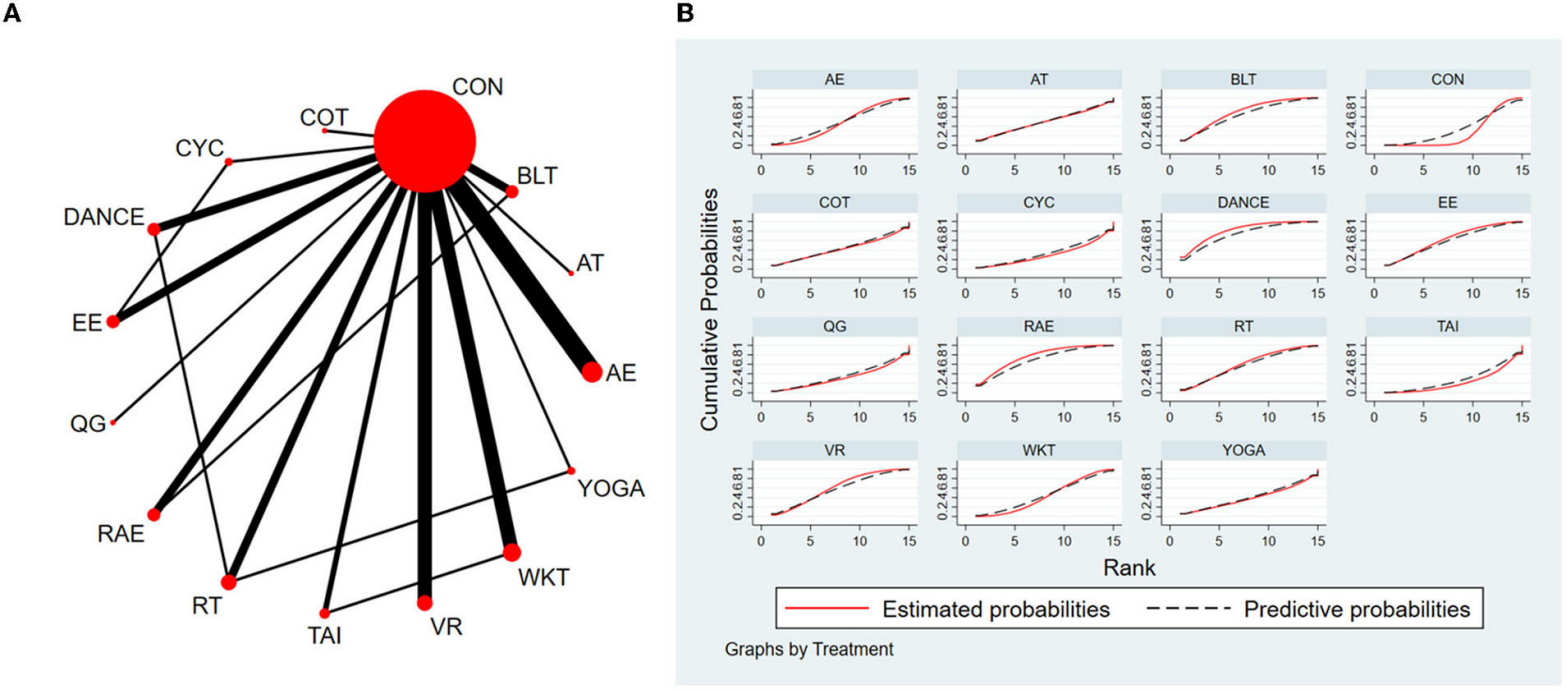
The NMA figure for BBS **(A)**. The SUCRA plot for BBS **(B)**. CON, Control Group; CYC, Cycling training; COT, Crossover training; BLT, Balance training; AT, Aerobic training; AE, Aquatic exercise; YOGA, Yoga; WKT, Walking training; VR, Virtual reality training; TAI, Tai Chi; RT, Resistance training; RAE, Rhythmical auditory exercise; QG, Qigong; EE, Exergaming exercise; DANCE, Dance.

**Figure 6:**
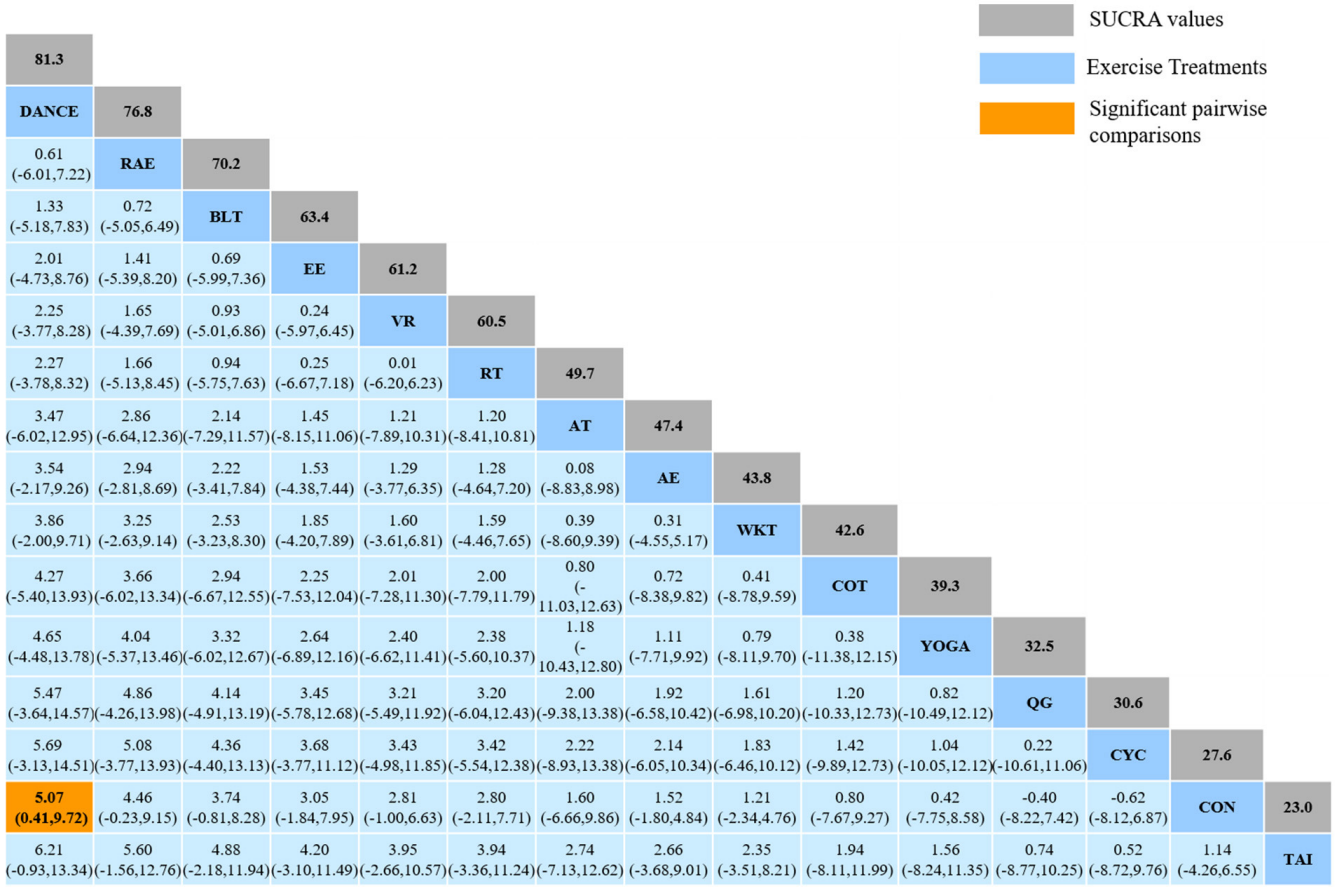
Relative effect sizes of efficacy on BBS according to network meta-analysis. CON, Control Group; CYC, Cycling training; COT, Crossover training; BLT, Balance training; AT, Aerobic training; AE, Aquatic exercise; YOGA, Yoga; WKT, Walking training; VR, Virtual reality training; TAI, Tai Chi; RT, Resistance training; RAE, Rhythmical auditory exercise; QG, Qigong; EE, Exergaming exercise; DANCE, Dance. Numbers in gray boxes were SUCRA values, which represented the rank of the intervention. Significant pairwise comparisons were highlighted in orange, in bold, and underlined.

The NMA for BBS were shown in Figure 5A. The probability ranking of the different exercise interventions in terms of improving BBS score was ranked first by Dance the SUCRA as shown in Figure 5B. The results of the Network meta-analysis showed that Dance (SUCRA = 81.3%), rhythmical auditory exercise (SUCRA = 76.8%), balance training (SUCRA = 70.2%), exergaming exercise (SUCRA = 63.4%), virtual reality training (SUCRA = 61.2%), resistance training (SUCRA = 60.5%), aerobic training (SUCRA = 49.7%), aquatic exercise (SUCRA = 47.4%), walking training (SUCRA = 43.8%), crossover training (SUCRA = 42.6%), Yoga (SUCRA = 39.3%), and cycling training (SUCRA = 30.6%) were higher than the control group (SUCRA = 27.6%) in increasing BBS score. The Dance (DANCE) was significantly superior to the named control group (CON) and underlying estimates of effect were presented (MD = 5.07, CI = 0.41, 9.72). The relative effect sizes of efficacy on BBS were shown in Figure 6.

For the consistency test, the node-splitting analysis was showed that all *p*-values for indirect and direct comparisons between studies were higher than 0.05, indicating a good convergence of the model and a better-iterated effect of enrolled studies. Details were shown in Supplementary Table 5.

#### 3.4.3. Mini-Balance Evaluation Systems Test

**Figure 7:**
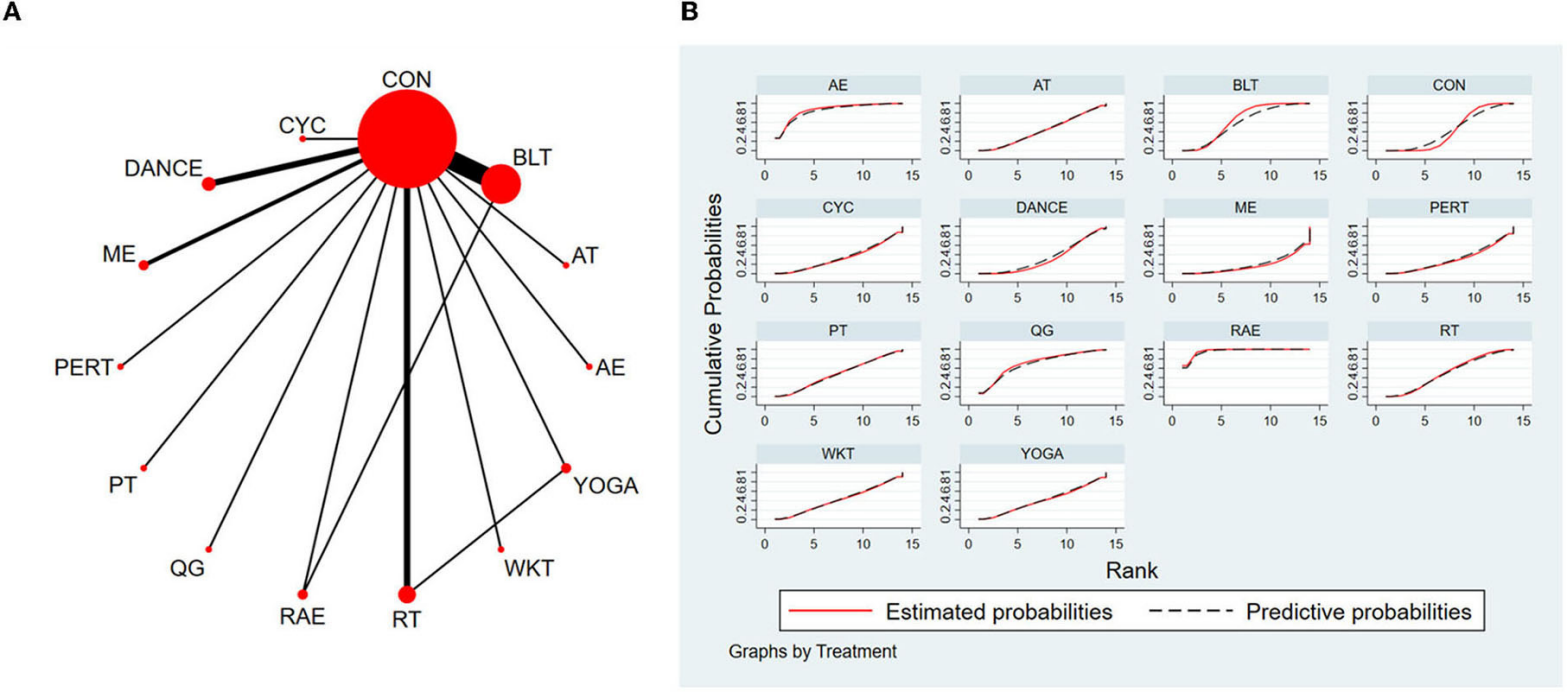
The NMA figure for Mini-BESTest **(A)**. The SUCRA plot for Mini-BESTest **(B)**. CON, Control Group; BLT, Balance training; AT, Aerobic training; AE, Aquatic exercise; YOGA, Yoga; WKT, Walking training; RT, Resistance training; RAE, Rhythmical auditory exercise; QG, Qigong; PT, Power training; PERT, Perturbation training; ME, Multiple exercises; DANCE, Dance; CYC, Cycling training.

**Figure 8:**
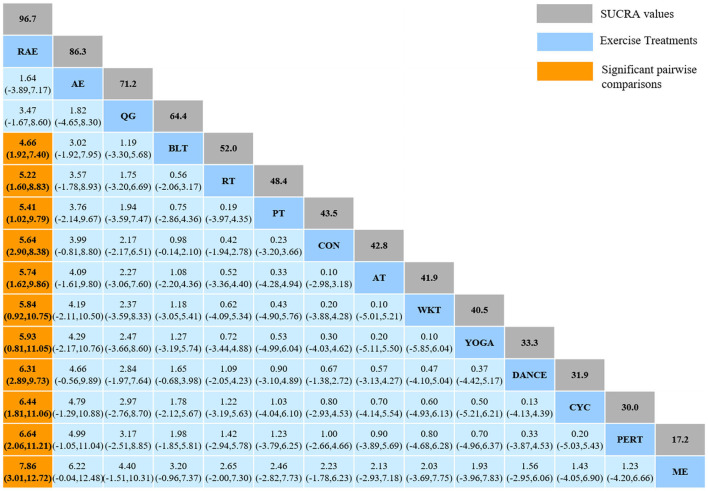
Relative effect sizes of efficacy on Mini-BESTest according to network meta-analysis. CON, Control Group; BLT, Balance training; AT, Aerobic training; AE, Aquatic exercise; YOGA, Yoga; WKT, Walking training; RT, Resistance training; RAE, Rhythmical auditory exercise; QG, Qigong; PT, Power training; PERT, Perturbation training; ME, Multiple exercises; DANCE, Dancing training; CYC, Cycling training. Numbers in gray boxes were SUCRA values, which represented the rank of the intervention. Significant pairwise comparisons were highlighted in orange, in bold, and underlined.

The NMA for Mini-BESTest were shown in Figure 7A. The probability ranking of the different exercise interventions in terms of improving Mini-BESTest scores was ranked first by rhythmical auditory exercise in the SUCRA which was shown in Figure 7B. The results of the Network meta-analysis showed that rhythmical auditory exercise (SUCRA = 95.6%), aquatic exercise (SUCRA = 86.3%), Qigong (SUCRA = 71.2%), balance training (SUCRA = 64.4%), resistance training (SUCRA = 52.0%) and power training (SUCRA = 48.4%) were higher than the control group in increasing Mini-BESTest score. The rhythmical auditory exercise (RAE) was significantly superior to the control group (CON) and underlying estimates of effect were presented (MD = 5.64, CI = 2.9, 8.38). Additionally, the RAE was significantly better than exercise intervention [ME (SMD = 7.86, CI = 3.01, 12.72), PERT (SMD = 6.64, CI = 2.06, 11.21), CYC (SMD = 6.44, CI = 1.81, 11.06), DANCE (SMD = 6.31, CI = 2.89, 9.73), YOGA (SMD = 5.93, CI = 0.81, 11.05), WKT (SMD = 5.84, CI = 0.92, 10.75), AT (SMD = 5.74, CI = 1.62, 9.86), PT (SMD = 5.41, CI = 1.02, 9.79), RT (SMD = 5.22, CI = 1.60, 8.83), BLT (SMD = 4.66, CI = 1.92, 7.40)]. The relative effect sizes of efficacy on Mini-BESTest were shown in Figure 8.

For the consistency test, the node-splitting analysis showed that all *p*-values for indirect and direct comparisons between studies were higher than 0.05, indicating a good convergence of the model and a better-iterated effect of enrolled studies. Details were shown in Supplementary Table 6.

### 3.5. Publication bias test

**Figure 9:**
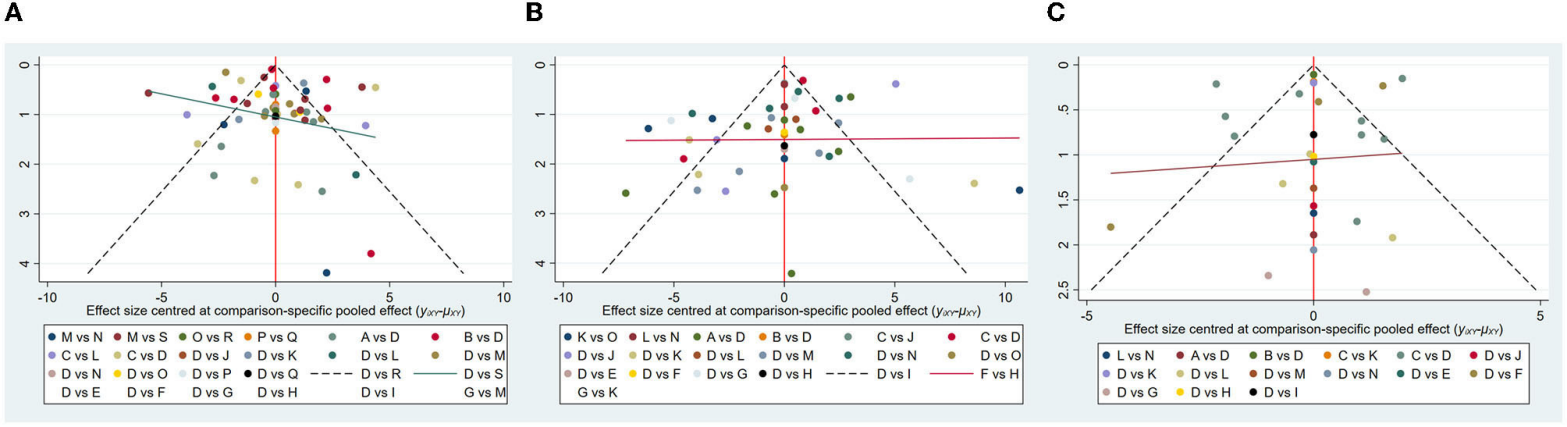
Funnel plot on publication bias of TUG **(A)**. BBS **(B)**. Mini-BESTest **(C)**.

The study formulated distinct funnel plots for all outcome indicators to check for probable publication bias. Visual inspection of the funnel plots did not reveal any significant publication bias (Wallace et al., 2009). Details were shown in Figure 9 for TUG (Figure 9A), BBS (Figure 9B), and MiniBest (Figure 9C). All *p*-values were > 0.05 and details of bias tests were shown in Supplementary material for TUG (Supplementary Table 7), BBS (Supplementary Table 8), and MiniBest (Supplementary Table 9).

## 4. Discussion

This network meta-analysis and systematic review investigated the effectiveness of various exercise interventions in reducing balance impairment in Parkinson's disease patients. A total of 60 studies, involving 20 distinct exercise regimens and 3,537 individuals with Parkinson's disease, were included. Our findings suggest that exergaming exercise was the optimal exercise intervention for lowering TUG test duration, Dance was the most effective intervention for improving BBS test scores, and rhythmic auditory exercise yielded the highest scores for Mini-BESTest. Notably, based on the combined ranking of these three test results in the study, we propose that rhythmic auditory exercise may be the most appropriate exercise intervention for enhancing postural balance in Parkinson's disease.

Regarding postural balance, we selected three balance tests of TUG, BBS, and Mini-BESTest corresponding to the index of postural balance, like dynamic balance, static balance, and body postural control. With increasing age, most individuals experience varying degrees of decline in muscle strength and flexibility, even in those who are otherwise healthy (Schaap et al., 2013; Yoo et al., 2020). The TUG test is a commonly used assessment tool to measure dynamic balance in older adults. In this study, exergaming exercise, rhythmic auditory exercise, and balance training were found to be highly effective in reducing TUG test times among individuals with Parkinson's disease. Moreover, the BBS evaluation serves as a comprehensive assessment of functional balance in Parkinson's disease patients, providing insights into their static balance by measuring their capacity to strategically relocate their center of gravity (Steffen et al., 2002; Pickenbrock et al., 2016). The Mini-BESTest is developed from the BESTest and focuses on anticipatory postural adjustments, reactive postural responses, sensory orientation, and gait stability with regards to postural control (Franchignoni et al., 2010; Padgett et al., 2012). It has been suggested that impaired postural balance in Parkinson's disease is caused by a variety of factors, necessitating a wide range of examinations to be performed by physical therapists (Jacobs et al., 2006). The Mini-BESTest incorporates multiple tests, which may account for its efficacy in identifying individuals with mild balance deficits. This study found that six different forms of intervention training resulted in improvements in Mini-BESTest scores compared to the control group. Of all the exercises evaluated, rhythmic auditory exercise was demonstrated to be the most beneficial.

As mentioned, Dance, exergaming exercise, and rhythmic auditory exercise were found to be the most promising exercise interventions for postural balance symptoms in patients with Parkinson's disease. The underlying mechanism for this effect may be explained in part by the two factors. Firstly, compared with other exercise interventions, these three exercise interventions are combined with auditory or visual stimulation. Consistent with earlier investigations (Wu et al., 2021; Hao et al., 2022; Yang et al., 2022), which also emphasized mobility and balance, the findings suggest that the combination of auditory or visual exercise may be more beneficial in facilitating interventions for patients with Parkinson's disease. According to Capato et al. (2020), the addition of rhythmic auditory stimulation may increase the effectiveness of rehabilitation training by enhancing attention and task prioritization, which would more effectively stimulate residual motor-cognitive function in Parkinson's disease patients than the conventional multimodal balance training. Similarly, other meta-analysis study supported that rhythmic auditory stimulation with conventional rehabilitation can enhance gait and motor performance in Parkinson's disease patients (Ghai et al., 2018). Exergaming exercise improve executive functions (prioritization processes) and visuospatial perception, which can increase the capacity to allocate cognitive resources in time and space (Mura et al., 2018). For people with Parkinson's disease, external cues may further boost activity in the putamen, a subcortical structure that facilitates movement, making up for the absence of dopaminergic stimulation (Nombela et al., 2013). Further, exergaming exercise improve patients' physical and visual memory functions by stimulating the subthalamic nucleus (Mollion et al., 2011). These benefits include assisting Parkinson's disease patients in creating complex coordinated movement sequences as well as improving general postural balance performance. Secondly, the significant change in postural balance may be attributed to multiple-skill learning. These three exercise interventions are intricate and demanding exercises. For example, Dance exercises involving non-periodic movements like starts, stops, rotations, side steps, and displacements in various directions benefit patients' training in body responsiveness and posture prediction (Repp and Su, 2013; Frisaldi et al., 2021). As a result, Parkinson's patients have improved their balance in various conditions, which leads to an increase in their scores on the BBS test. Our findings indicate that Dance has a significant beneficial effect on the balance of Parkinson's disease patients compared to other forms of exercise and that there is a statistically significant difference between the experimental group and the control group, which is also in line with the findings of previous research (Hidalgo-Agudo et al., 2020; Hao et al., 2022).

## 5. Strengths and limitations

First and foremost, this study is the most comprehensive and systematic comparative meta-analysis of the effects of exercises on Parkinson's disease patients. We included 60 studies and 3,537 patients, a considerable sample size. We also expanded on the original review of postural balance improvement in people with Parkinson's disease by including four novel interventions. They were exergaming exercise, rhythmic auditory exercise, crossover training, and virtual reality exercises, which were compared with other interventions, to provide a new and comprehensive evidence-based recommendation. Overall, the study has some clinical implications. Exergaming exercise, Dance, and rhythmic auditory exercise significantly improve postural balance in Parkinson's disease. Furthermore, doctors can promote exercise as an excellent non-pharmacological intervention for managing Parkinson's disease.

Our study also has some limitations in ordinary with the study on which it is based. Four studies were judged at high risk of selection bias and other biases, respectively. The quality of risk studies potentially threatened the validity of our study. Although we adjusted for study heterogeneity when including these original findings, variability between studies could not be avoided (e.g., the gender ratio of participants and the proportion of regional studies).

## 6. Conclusion

This study proposed the adoption of specific exercise types for improving the postural balance of patients with Parkinson's disease. Exergaming exercise was recommended for those seeking to enhance their dynamic balance, while Dance and rhythmic auditory exercise were suggested for individuals looking to improve their static balance and postural control. In general, exercise prescriptions for Parkinson's patients include exergaming exercise, Dance, and rhythmic auditory exercise, which have been shown to be effective in enhancing postural balance.

## Data availability statement

The original contributions presented in the study are included in the article/Supplementary material, further inquiries can be directed to the corresponding authors.

## Author contributions

DW and YG conceived and designed the study. DW and WC contributed to the literature research, the study selection, and the data extraction. DW and ZH performed the quality assessment and analyzed the data. DW was the funding acquisition and the major contributor in interpreting the results and writing the original manuscript. All authors contributed to the article and approved the submitted version.
