# Supplementary material for: Effectiveness of different exercises in improving postural balance among Parkinson's disease patients: a systematic review and network meta-analysis
Source: Front Aging Neurosci. 2023 Jul 17;15:1215495. doi: 10.3389/fnagi.2023.1215495 (PMC10388555; doi:10.3389/fnagi.2023.1215495)
Supplement: Supplementary file 1 [file Data_Sheet_1.doc]

**Supplementary Content**

Literature Search Strategy ....................................................................................................................................... 2

Table 1: Details of the Literature Search Strategy .................................................................................................. 2

1. PubMed (Mar 30, 2023) ................................................................................................................................ 2
2. Cochrane Library (Mar 31, 2023).................................................................................................................. 4
3. Embase (Mar 31, 2023) ................................................................................................................................ 5
4. Web of Science (Mar 31, 2023) .................................................................................................................... 6
5. PsycINFO (Mar 31, 2023) ............................................................................................................................ 7

Table 2: Definitions of exercise interventions and controls ................................................................................... 8

Table 3: Characteristics of the studies included in the meta-analysis .................................................................... 10

Table 4: Details of consistency test for TUG ......................................................................................................... 22

Table 5: Details of consistency test for BBS .......................................................................................................... 23

Table 6: Details of consistency test for Mini-BESTest .......................................................................................... 24

Table 7: Details of bias test for TUG ..................................................................................................................... 25

Table 8: Details of bias test for BBS ...................................................................................................................... 26

Table 9: Details of bias test for Mini-BESTest ....................................................................................................... 27

**Literature Search Strategy**

**Table 1: Details of the Literature Search Strategy**

(A) PubMed (Mar 30, 2023)

| **Search** | **Query** | **Items found** |
| --- | --- | --- |
| #1 | "Parkinson Disease"[Mesh] | 8060 |
| #2 | (((((Parkinson disease[Title/Abstract])OR Parkinson′s disease[Title/Abstract]) OR idiopathic Parkinson′s disease[Title/Abstract]) OR lewy body Parkinson′s disease[Title/Abstract]) OR primary Parkinsonism[Title/Abstract]) OR paralysis agitans[Title/Abstract] | 11,605 |
| #3 | #1OR#2 | 12,912 |
| #4 | "Exercise"[Mesh] | 24,290 |
| #5 | ((((((((((((((((((((((((Exercises[Title/Abstract]) OR (Physical Activity[Title/Abstract])) OR (Activities, Physical[Title/Abstract])) OR (Activity, Physical[Title/Abstract])) OR (Physical Activities[Title/Abstract])) OR (Exercise, Physical[Title/Abstract])) OR (Exercises, Physical[Title/Abstract])) OR (Physical Exercise[Title/Abstract])) OR (Physical Exercises[Title/Abstract])) OR (Acute Exercise[Title/Abstract])) OR (Acute Exercises[Title/Abstract])) OR (Exercise, Acute[Title/Abstract])) OR (Exercises, Acute[Title/Abstract])) OR (Exercise, Isometric[Title/Abstract])) OR (Exercises, Isometric[Title/Abstract])) OR (Isometric Exercises[Title/Abstract])) OR (Isometric Exercise[Title/Abstract])) OR (Exercise, Aerobic[Title/Abstract])) OR (Aerobic Training[Title/Abstract])) OR (Aerobic Trainings[Title/Abstract])) OR (Exercises, Aerobic[Title/Abstract])) OR (Exercise Training[Title/Abstract])) OR (Exercise Trainings[Title/Abstract])) OR (Training, Exercise[Title/Abstract])) OR (Trainings, Exercise[Title/Abstract]) | 28,671 |
| #6 | #4 OR #5 | 41,782 |
| #7 | #3 AND #6 | 3118 |

**(B) Cochrane Library** **(Mar 31, 2023)**

| **Search** | **Query** | **Items found** |
| --- | --- | --- |
| #1 | MeSH descriptor: [Parkinson Disease] explode all trees | 6099 |
| #2 | (Parkinson disease):ti,ab,kw OR (Parkinson′s disease):ti,ab,kw OR (idiopathic Parkinson′s disease):ti,ab,kw OR (lewy body Parkinson′s disease):ti,ab,kw OR (primary Parkinsonism):ti,ab,kw OR (paralysis agitans):ti,ab,kw | 12,396 |
| #3 | #1 OR #2 | 12,396 |
| #4 | MeSH descriptor: [Exercise] explode all trees | 37,900 |
| #5 | (Exercises):ti,ab,kw OR (Physical Activity):ti,ab,kw OR (Activities, Physical):ti,ab,kw OR (Activity, Physical):ti,ab,kw OR (Physical Activities):ti,ab,kw OR (Exercise, Physical):ti,ab,kw OR (Exercises, Physical):ti,ab,kw OR (Physical Exercise):ti,ab,kw OR (Physical Exercises):ti,ab,kw OR (Acute Exercise):ti,ab,kw OR (Acute Exercises):ti,ab,kw OR (Exercise, Acute):ti,ab,kw OR (Exercises, Acute):ti,ab,kw OR (Exercise, Isometric):ti,ab,kw OR (Exercises, Isometric):ti,ab,kw | 169,265 |
| #6 | #4 OR #5 | 172,960 |
| #7 | #3 AND #6 | 2092 |

(C) Embase (Mar 31, 2023)

| **Search** | **Query** | **Items found** |
| --- | --- | --- |
| #1 | 'parkinson disease'/exp | 190,479 |
| #2 | 'parkinson disease':ab,ti OR 'parkinson′s disease':ab,ti OR 'idiopathic parkinson′s disease':ab,ti OR 'lewy body parkinson′s disease':ab,ti OR 'primary parkinsonism':ab,ti OR 'paralysis agitans':ab,ti | 163,645 |
| #3 | #1 OR #2 | 213,975 |
| #4 | 'exercise'/exp | 430,815 |
| #5 | exercises:ab,ti OR 'physical activity':ab,ti OR 'activities, physical':ab,ti OR 'activity, physical':ab,ti OR 'physical activities':ab,ti OR 'exercise, physical':ab,ti OR 'exercises, physical':ab,ti OR 'physical exercise':ab,ti OR 'physical exercises':ab,ti OR 'acute exercise':ab,ti OR 'acute exercises':ab,ti OR 'exercise, acute':ab,ti OR 'exercises, acute':ab,ti OR 'exercise, isometric':ab,ti OR 'exercises, isometric':ab,ti OR 'isometric exercises':ab,ti OR 'isometric exercise':ab,ti OR 'exercise, aerobic':ab,ti OR 'aerobic exercise':ab,ti OR 'aerobic exercises':ab,ti OR 'exercises, aerobic':ab,ti OR 'exercise training':ab,ti OR 'exercise trainings':ab,ti OR 'training, exercise':ab,ti OR 'trainings, exercise':ab,ti | 388,159 |
| #6 | #4 OR #5 | 633,820 |
| #7 | #3 AND #6 | 5430 |

(D) Web of Science (Mar 31, 2023)

| **Search** | **Query** | **Items found** |
| --- | --- | --- |
| #1 | Parkinson Disease (Topic) and Preprint Citation Index (Exclude – Database) | 262,830 |
| #2 | Parkinson disease (Topic) or parkinson disease (Topic) or parkinson′s disease (Topic) or idiopathic parkinson′s disease (Topic) or lewy body parkinson′s disease (Topic) or primary parkinsonism (Topic) or paralysis agitans (Topic) or Preprint Citation Index (Topic) and Preprint Citation Index (Exclude – Database) | 263,849 |
| #3 | #1 OR #2 and Preprint Citation Index (Exclude – Database) | 263,849 |
| #4 | TS=(Exercise) and Preprint Citation Index (Exclude – Database) | 1,066,809 |
| #5 | Exercises (Topic) or Physical Activity (Topic) or Activities, Physical (Topic) or Activity, Physical (Topic) or Physical Activities (Topic) or Exercise, Physical (Topic) or Exercises, Physical (Topic) or Physical Exercise (Topic) or Physical Exercises (Topic) or Acute Exercise (Topic) or Acute Exercises (Topic) or Exercise, Acute (Topic) or Exercises, Acute (Topic) or Exercise, Isometric (Topic) or Exercises, Isometric (Topic) and Preprint Citation Index (Exclude – Database) | 1,860,227 |
| #6 | #4 OR #5 and Preprint Citation Index (Exclude – Database) | 1,860,227 |
| #7 | #3 AND #6 and Preprint Citation Index (Exclude – Database) | 7924 |

(E) PsycINFO (Mar 31, 2023)

| **Search** | **Query** | **Items found** |
| --- | --- | --- |
| #1 | MJ Parkinson Disease | 23,441 |
| #2 | DE Parkinson disease OR DE Parkinson′s disease OR DE idiopathic Parkinson′s disease OR DE lewy body Parkinson′s disease OR DE primary Parkinsonism OR DE paralysis agitans | 27,662 |
| #3 | (DE Parkinson disease OR DE Parkinson′s disease OR DE idiopathic Parkinson′s disease OR DE lewy body Parkinson′s disease OR DE primary Parkinsonism OR DE paralysis agitans) AND (S1 OR S2) | 27,662 |
| #4 | MJ Exercise | 23,567 |
| #5 | DE Exercises OR DE Physical Activity OR DE Activities, Physical OR DE Activity, Physical OR DE Physical Activities OR DE Exercise, Physical OR DE Exercises, Physical OR DE Physical Exercise OR DE Physical Exercises OR DE Acute Exercise OR DE Acute Exercises OR DE Exercise, Acute OR DE Exercises, Acute OR DE Exercise, Isometric OR DE Exercises, Isometric OR DE Preprint Citation Index | 25,804 |
| #6 | S4 OR S5 | 25,804 |
| #7 | S3 AND S6 | 117 |

**Table 2: Definitions of exercise interventions and controls.**

| **Type** | **Definition** |
| --- | --- |
| Aerobic training | Exercise intensity was monitored to maintain a heart rate consistent with 50–80% of max VO_2_. The exercise modalities included walking, recycling, stretching, and physical fitness. |
| Aquatic exercise | Exercise performed in the water. |
| Balance training | Balance exercises concluded with static and dynamic balance exercises under different sensory conditions. |
| Cycling training | Exercise using a special stationary exercise bicycle with a weighted flywheel in an indoor setting. |
| Crossover training | Crossover is a device that simulates the movement of a skater, broadening the support base and improving the functionality of paravertebral muscles. |
| Dance | Exercise form consisting of sequences of movement, either improvised or purposefully selected. The study included Folk, Jazz, Waltz, Foxtrot,, and Tango styles. |
| Exergaming exercise | Exercise performed by video games. |
| Multiple exercises | The intervention included two or more specific types of exercise, like boxing, walking, balance, stretching, and upper limb exercise. |
| Power training | Exercise concentric muscle contractions are performed as fast as possible at light-to-moderate loads, based on the proposition that some aspects of physical function (e.g., chair rise, stair climb). |
| Perturbation training | Perturbations by applying external forces to the body, or sudden mediolateral or anterior-posterior belt translations. |
| Qigong | Exercise typically focuses on movement, breathing, and meditation, to integrate the body, breath, and mind adjustments into one. |
| Resistance training | Resistance training, namely isometric training and isokinetic training, is a kind of muscle strength training method to maintain a constant speed of movement. |
| Resistance training with instability | Exercise incorporates dual-task exercises with challenging and complex (only free weights exercises performed on unstable devices) balance exercises. |
| Rhythmical auditory exercise | Exercises equipped with rhythmic auditory stimulation to improve physical functions for Parkinson's disease by harnessing the innate internal timing process through external cues. |
| Tai Chi | Exercise following traditional Chinese martial art principles to improve strength, balance, and physical function. |
| Traditional exercise | This program included stretching exercises, strengthening exercises, and balance exercises in each session. |
| Virtual reality training | Physical exercise is interactively combined with cognitive stimulation in a virtual environment. |
| Walking training | Walking training included ordinary walking and Nordic walking. Walking training practiced by using land-based modes or specially designed walking poles like ski poles. |
| Yoga | Exercise that combines physical elements with traditional yoga principles. |
| Control | The control group included wait-list control (no intervention provided) and active control (usual care or rehabilitation training). |

**Table 3: Characteristics of the studies included in the meta-analysis**

| Author | Year | Population | Age(mean+SD) | Total/male/female | Intervention | Control | Outcome |
| --- | --- | --- | --- | --- | --- | --- | --- |
| Moraes Filho | 2020 | Parkinson disease  Hoehn & Yahr1-3 | T:64.7(1.8)  C:64.4(3.7) | T:25/20/5  C:15/10/5 | Resistance Training  Length of Intervention: 9 weeks  Freq: 2 times a week  Duration: NA | CON | TUG |
| Silva | 2018 | Parkinson disease  Hoehn & Yahr1-3 | T: 63.12(13.61)  C: 64.23(13.45) | T: 14/8/6  C: 11/6/5 | Aquatic Exercise  Length of Intervention: 10 weeks  Freq: 2 times a week  Duration: 40 min | CON | TUG, BBS |
| Mak | 2021 | Parkinson disease  Hoehn & Yahr1-3 | T: 61.9(6.4)  C: 62.7(7.2) | T: 33/22/11  C: 31/22/9 | Walking Training  Length of Intervention: 10 weeks  Freq: 1 times a week  Duration: 90 min | ACT | Mini-BEST, TUG |
| Laio | 2014 | Parkinson disease  Hoehn & Yahr1-3 | T1: 67.3(7.1)  T2: 65.1(6.7)  C: 64.6(8.3) | T1: 12/5/7  T2: 12/6/6  C: 12/6/6 | 1. VR Training 2. Traditional Training   Length of Intervention: 6 weeks  Freq: 2 times a week  Duration: 45 min | CON | TUG |
| Li | 2022 | Parkinson disease  Hoehn & Yahr1-2.5 | T1: 62.7(5.51)  T2: 61.9(5.64)  C: 61.9(6.76) | T1: 32/17/15  T2: 31/22/9  C: 32/19/13 | 1. Tai Chi 2. Walking Training   Length of Intervention: 12 MOs  Freq: 2 times a week  Duration: 60 min | CON | TUG, BBS |
| Elisa | 2017 | Parkinson disease  Hoehn & Yahr1-2.5 | T: 69.9(7.4)  C: 70(6.4) | T: 8/4/4  C: 8/3/5 | Cycling Training  Length of Intervention: 10 sessions  Freq: 2 times a week  Duration: 40 min | ACT | TUG, BBS |
| Oliveira | 2020 | Parkinson disease  Hoehn & Yahr1-3 | T: 65.5(2.16)  C: 68.33(0.413) | T: 9/8/1  C: 10/9/1 | Aquatic Exercise  Length of Intervention: 4 weeks  Freq: 2 times a week  Duration: 60 min | ACT | TUG, BBS |
| Carla | 2016 | Parkinson disease  Hoehn & Yahr1-3 | T1: 64.1(9.1)  T2: 64.2(10.6)  C: 64.2(8.3) | T1: 13/10/3  T2: 13/10/3  C: 13/9/4 | 1. Resistance Training(RT) 2. RT with instability   Length of Intervention: 12 weeks  Freq: 2 times a week  Duration: 60 min | CON | TUG |
| Irene | 2015 | Parkinson disease  Hoehn & Yahr1-3 | T: 59.4(9)  C: 62.6(8.9) | T: 41/25/16  C: 39/21/18 | Balance Training  Length of Intervention: 4 weeks  Freq: 2 times a week  Duration: 60 min | ACT | TUG |
| Yang | 2021 | Parkinson disease  Hoehn & Yahr1-3 | T1: 67(20)  T2: 65(28)  C: 66(24) | T1: 30/10/20  T2: 30/15/15  C: 28/14/14 | 1. Early-Exercise 2. Late-Exercise   Length of Intervention: 18 MOs  Freq: 2 times a week  Duration: 60 min | CON | Mini-BEST, TUG |
| Pereira-Pedro | 2022 | Parkinson disease  Hoehn & Yahr1-3 | T: 70.50(9.24)  C: 65.43(5.22) | T: 8/6/2  C: 7/6/1 | Cycling Training  Length of Intervention: 7 weeks  Freq: 2 times a week  Duration: 20 min | ACT | TUG |
| Bang | 2016 | Parkinson disease  Hoehn & Yahr1-3 | T: 58.30(7.71)  C: 60.6(6.74) | T: 10/5/5  C: 10/4/6 | Walking Training  Length of Intervention: 4 weeks  Freq: 5 times a week  Duration: 60 min | ACT | TUG, BBS |
| Frazzitta | 2015 | Parkinson disease  Hoehn & Yahr1-3 | T: 65(8.8)  C: 66.6(10.0) | T: 30/17/13  C: 30/13/17 | Crossover Training  Length of Intervention: 4 weeks  Freq: 6 times a week  Duration: 35 min | ACT | TUG, BBS |
| Liu | 2022 | Parkinson disease  Hoehn & Yahr1-3 | T: 70.93(7.23)  C: 64.79(5.86) | T: 14/8/6  C: 14/8/6 | Balance Training  Length of Intervention: 8 weeks  Freq: 2 times a week  Duration: 60 min | ACT | Mini-BEST, TUG |
| Terrens | 2019 | Parkinson disease  Hoehn & Yahr1-3 | T1: 74.1(6.6)  T2: 65.6(7.7)  C: 76.4(7.4) | T1: 11/10/1  T2: 10/7/3  C: 9/7/2 | 1. Aquatic Exercise(Halliwick) 2. Aquatic Exercise   Length of Intervention: 18 MOs  Freq: 2 times a week  Duration: 60 min | ACT | Mini-BEST, BBS |
| Kolk | 2019 | Parkinson disease  Hoehn & Yahr1-2 | T: 59.3(8.3)  C: 59.4(9.3) | T: 65/42/23  C: 65/38/27 | Aerobic Training  Length of Intervention: 6 MOs  Freq: 3 times a week  Duration: 45 min | ACT | Mini-BEST, TUG |
| Marialuisa | 2017 | Parkinson disease  Hoehn & Yahr1-3 | T: 67.45(7.18)  C: 69.84(9.41) | T: 38/23/15  C: 38/28/10 | VR Training  Length of Intervention: 7 weeks  Freq: 3 times a week  Duration: 50 min | ACT | BBS |
| Yang | 2015 | Parkinson disease  Hoehn & Yahr1-3 | T: 72.4(8.4)  C: 75.4(5.3) | T: 11/4/7  C: 12/5/7 | VR Training  Length of Intervention: 6 weeks  Freq: 2 times a week  Duration: 50 min | ACT | TUG, BBS |
| Solla | 2019 | Parkinson disease  Hoehn & Yahr1-3 | T: 67.8(5.9)  C: 67.1(6.3) | T: 10/6/4  C: 10/7/3 | Dancing(Sardinian Folk)  Length of Intervention: 12 weeks  Freq: 2 times a week  Duration: 90 min | CON | TUG, BBS |
| Kashif | 2022 | Parkinson disease  Hoehn & Yahr1-3 | T: 63.86(4.57)  C: 62.32(4.61) | T: 22/13/9  C: 22/12/10 | VR Training  Length of Intervention: 12 weeks  Freq: 3 times a week  Duration: 60 min | ACT | BBS |
| ATAN | 2019 | Parkinson disease  Hoehn & Yahr2-4 | T1: 68.6(8.2)  T2: 72.2(7.9)  C: 69.7(8) | T1: 10/6/4  T2: 10/6/4  C: 10/3/7 | Resistance Training(ATAN)  Length of Intervention: 6 weeks  Freq: 5 times a week  Duration: 30 min | CON | BBS |
| Fatih Soke | 2019 | Parkinson disease  Hoehn & Yahr1-3 | T: 57.7(8.1)  C: 56.2(8.7) | T: 14/10/4  C: 12/8/4 | Aerobic Training  Length of Intervention: 7 weeks  Freq: 3 times a week  Duration: 50 min | ACT | TUG, BBS |
| Pazzaglia | 2019 | Parkinson disease  UPDRS III | T: 72(7)  C: 70(10) | T: 25/18/7  C: 26/17/9 | VR Training  Length of Intervention: 6 weeks  Freq: 3 times a week  Duration: 40 min | ACT | BBS |
| Tollár | 2019 | Parkinson disease  Hoehn & Yahr1-3 | T1: 70.0(4.69)  T2: 70.6(4.10)  C: 67.5(4.28) | T1: 25/12/13  T2: 25/11/14  C: 24/13/11 | 1. Exergaming Exercise 2. Cycling Training   Length of Intervention: 5 weeks  Freq: 5 times a week  Duration: 60 min | CON | BBS |
| Palamara | 2017 | Parkinson disease  Hoehn & Yahr1-3.5 | T: 70.9(5.7)  C: 70.8(5.3) | T: 17/9/8  C: 17/11/6 | Aquatic Exercise  Length of Intervention: 12 weeks  Freq: 3 times a week  Duration: 60 min | ACT | TUG, BBS |
| Shih | 2016 | Parkinson disease  Hoehn & Yahr1-2.5 | T: 67.5(9.96)  C: 68.8(9.67) | T: 10/9/1  C: 10/7/3 | Exergaming Exercise  Length of Intervention: 8 weeks  Freq: 2 times a week  Duration: 50 min | ACT | TUG, BBS |
| Hashimoto | 2015 | Parkinson disease  Hoehn & Yahr1-4 | T1: 67.9(7.0)  T2: 62.7(14.9)  C: 69.7(4.0) | T1: 15/3/12  T2: 17/2/15  C: 14/7/7 | 1. Dancing(Jazz) 2. Resistance Training   Length of Intervention: 8 weeks  Freq: 2 times a week  Duration: 50 min | CON | TUG, BBS |
| Harro | 2014 | Parkinson disease  Hoehn & Yahr1-3 | T: 67.3(10.88)  C: 64.9(8.98) | T: 10/8/2  C: 10/5/5 | Rhythmic Auditory Exercise  Length of Intervention: 6 weeks  Freq: 3 times a week  Duration: 30 min | ACT | BBS |
| Vivas | 2011 | Parkinson disease  Hoehn & Yahr1-3 | T: 68.33(6.92)  C: 65.67(3.67) | T: 6/4/2  C: 6/3/3 | Aquatic Exercise  Length of Intervention: 4 weeks  Freq: 2 times a week  Duration: 40 mine | TRD | TUG, BBS |
| Smania | 2010 | Parkinson disease  Hoehn & Yahr1-3 | T: 67.64(7.41)  C: 67.26(7.18) | T: 28/14/14  C: 27/15/12 | Balance Training  Length of Intervention: 7 weeks  Freq: 3 times a week  Duration: 50 mine | ACT | BBS |
| Feng | 2019 | Parkinson disease  Hoehn & Yahr1-3.5 | T: 67.47(4.79)  C: 66.93(4.64) | T: 14/8/7  C: 14/9/6 | VR Training  Length of Intervention: 12 weeks  Freq: 5 times a week  Duration: 45 min | ACT | TUG, BBS |
| Capato | 2020 | Parkinson disease  Hoehn & Yahr1-3 | T1: 74(8)  T2: 67(13)  C: 73(10) | T1: 56/27/29  T2: 50/32/18  C: 48/29/19 | 1. Rhythmic Auditory Exercise 2. Balance Training   Length of Intervention: 6 weeks  Freq: 3 times a week  Duration: 30 min | ACT | Mini-BEST, TUG, BBS |
| CRUZ | 2017 | Parkinson disease  Hoehn & Yahr2-3 | T: 66.80(5.27)  C: 67.53(9.89) | T: 15/6/9  C: 15/7/8 | Aquatic Exercise  Length of Intervention: 10 weeks  Freq: 2 times a week  Duration: 45 mine | ACT | TUG, BBS |
| Ni | 2016 | Parkinson disease  Hoehn & Yahr1-3 | T1: 71.6(6.6)  T2: 71.2(6.5)  C: 74.9(8.3) | T1: 14/9/5  T2: 13/11/2  C: 10/4/6 | 1. Resistance Training 2. Yoga   Length of Intervention: 12 weeks  Freq: 2 times a week  Duration: 60 min | CON | Mini-BEST, TUG, BBS |
| Kurt | 2017 | Parkinson disease  Hoehn & Yahr2-3 | T: 62.41(6.76)  C: 63.61(7.18) | T: 20/9/11  C: 20/7/13 | Aquatic Exercise  Length of Intervention: 12 weeks  Freq: 2 times a week  Duration: 20 mine | ACT | TUG, BBS |
| Zhang | 2015 | Parkinson disease  Hoehn & Yahr1-3 | T: 66(11.80)  C: 64.35(10.53) | T: 20/13/7  C: 20/11/9 | Tai Chi  Length of Intervention: 12 weeks  Freq: 2 times a week  Duration: 20 mine | ACT | TUG, BBS |
| Xiao | 2015 | Parkinson disease  Hoehn & Yahr1-2.5 | T: 68.17(2.17)  C: 66.52(2.13) | T: 48/33/15  C: 48/34/14 | Qigong (Baduanjin)  Length of Intervention: 6 MOs  Freq: 4 times a week  Duration: 20 mine | ACT | TUG, BBS |
| Hackney | 2009 | Parkinson disease  Hoehn & Yahr1-2.5 | T1: 66.8(2.4)  T2: 68.2(1.4)  C: 66.5(2.8) | T1: 17/11/6  T2: 14/11/3  C: 17/12/5 | 1. Dancing (Waltz/ Foxtrot) 2. Dancing (Tango)   Length of Intervention: 13 weeks  Freq: 2 times a week  Duration: 60 mine | CON | TUG, BBS |
| Monteiro | 2016 | Parkinson disease  Hoehn & Yahr1-3 | T: 64.9(10.2)  C: 70.5(5.8) | T: 16/13/3  C: 17/7/10 | Walking Training  Length of Intervention: 6 weeks  Freq: 2 times a week  Duration: 40 min | ACT | TUG, BBS |
| SPINA | 2021 | Parkinson disease  Hoehn & Yahr1-2.5 | T: 68(6.9)  C: 67.27(4.85) | T: 11/5/6  C: 11/4/7 | Robotic Balance Training  Length of Intervention: 6 weeks  Freq: 2 times a week  Duration: 40 min | ACT | Mini-BEST, BBS |
| Calabrò | 2019 | Parkinson disease  Hoehn & Yahr2-3 | T: 70(8)  C: 73(8) | T: 20/9/11  C: 20/6/14 | Rhythmic Auditory Exercise  Length of Intervention: 8 weeks  Freq: 5 times a week  Duration: 120 min | ACT | TUG, BBS |
| Kim | 2019 | Parkinson disease  Hoehn & Yahr1-4 | T: 71(7.7)  C: 73(7.7) | T: 238/147/91  C: 236/119/117 | Balance Training  Length of Intervention: 6 MOs  Freq: 2 times a week  Duration: 60 min | CON | Mini-BEST |
| Araceli | 2018 | Parkinson disease  Hoehn & Yahr2-3 | T: 74.2(5.8)  C: 75.4(6.5) | T: 238/147/91  C: 239/119/117 | Resistance Training  Length of Intervention: 8 weeks  Freq: 2 times a week  Duration: 60 min | ACT | Mini-BEST |
| SANTOS | 2017 | Parkinson disease  Hoehn & Yahr1-3 | T: 67(7.9)  C: 68.5(6.5) | T: 14/11/3  C: 12/7/5 | Balance Training  Length of Intervention: 12 weeks  Freq: 2 times a week  Duration: 60 min | ACT | Mini-BEST |
| Christian | 2015 | Parkinson disease  Hoehn & Yahr1-3 | T: 75.7(5.5)  C: 75.7(7.2) | T: 17/12/5  C: 15/9/6 | Resistance Training  Length of Intervention: 8 weeks  Freq: 2 times a week  Duration: 60 min | ACT | Mini-BEST,TUG |
| Giardini | 2018 | Parkinson disease  Hoehn & Yahr1-3 | T: 68(8)  C: 73.1(15.6) | T: 17/13/4  C: 15/8/7 | Balance Training  Length of Intervention: 4 weeks  Freq: 3 times a week  Duration: 45 min | ACT | Mini-BEST, TUG |
| Silvia | 2015 | Parkinson disease  Hoehn & Yahr1-3 | T: 68(8)  C: 64.3(8.1) | T: 18/12/6  C: 15/7/8 | Dancing (Tango)  Length of Intervention: 4 weeks  Freq: 3 times a week  Duration: 45 min | CON | Mini-BEST,TUG |
| David | 2015 | Parkinson disease  Hoehn & Yahr2-3 | T: 72.9(6)  C: 73.6(5.3) | T: 47/28/19  C: 44/23/21 | Balance Training  Length of Intervention: 10 weeks  Freq: 3 times a week  Duration: 60 min | CON | Mini-BEST |
| Steib | 2017 | Parkinson disease  Hoehn & Yahr1-3.5 | T: 67.6(8.2)  C: 62.5(7.9) | T: 18/11/7  C: 20/16/4 | Walking Training  Length of Intervention: 8 weeks  Freq: 2 times a week  Duration: 35 min | ACT | Mini-BEST,TUG |
| Arcolin | 2015 | Parkinson disease  Hoehn & Yahr1.5-3 | T: 67.8(8.8)  C: 68.3(8.3) | T: 13/6/7  C: 16/9/7 | Cycling Training  Length of Intervention: 3 weeks  Freq: 5 times a week  Duration: 60 min | ACT | Mini-BEST,TUG |
| Shanahan | 2017 | Parkinson disease  Hoehn & Yahr1-2.5 | T: 69(10)  C: 69(8) | T: 20/13/7  C: 21/13/8 | Dancing  Length of Intervention: 10 weeks  Freq: 3 times a week  Duration: 20 min | CON | Mini-BEST |
| Irene | 2015 | Parkinson disease  Hoehn & Yahr2-3 | T: 60.2(9)  C: 61.9(8.5) | T: 32/19/13  C: 36/20/16 | Balance Training  Length of Intervention: 8 weeks  Freq: 2 times a week  Duration: 120 min | ACT | Mini-BEST,TUG |
| Frisaldi | 2021 | Parkinson disease  Hoehn & Yahr1-2 | T: 61.21(7.8)  C: 60.68(6.34) | T: 19/13/6  C: 19/10/9 | Dancing  Length of Intervention: 5 weeks  Freq: 3 times a week  Duration: 60 min | ACT | Mini-BEST,TUG |
| Cherup | 2021 | Parkinson disease  Hoehn & Yahr1-3 | T: 70.19(9.06)  C: 68.63(10.54) | T: 17/9/8  C: 18/11/7 | Power Training  Length of Intervention: 12 weeks  Freq: 3 times a week  Duration: 60 min | ACT | Mini-BEST,TUG |
| Yuan | 2020 | Parkinson disease  Hoehn & Yahr1-3 | T: 67.8(5.5)  C: 66.5(8.8) | T: 10/2/10  C: 12/9/3 | Exergaming Exercise  Length of Intervention: 6 weeks  Freq: 3 times a week  Duration: 30 min | CON | BBS |
| Song | 2018 | Parkinson disease  Hoehn & Yahr1-3 | T: 68(7)  C: 65(7) | T: 28/12/16  C: 25/5/20 | VR Training  Length of Intervention: 12 weeks  Freq: 3 times a week  Duration: 15 min | CON | TUG |
| Li | 2022 | Parkinson disease  Hoehn & Yahr1-3 | T: 67.57(3.95)  C: 70(5.59) | T: 20/13/7  C: 20/16/4 | Qigong Training  Length of Intervention: 12 weeks  Freq: 2 times a week  Duration: 90 min | ACT | Mini-BEST,TUG |
| Sacheli | 2019 | Parkinson disease  Hoehn & Yahr1-3 | T: 66.76(5.9)  C: 67.85(8.5) | T: 20/13/7  C: 15/9/6 | Aerobic Training  Length of Intervention: 12 weeks  Freq: 3 times a week  Duration: 60 min | CON | TUG |
| Strand | 2021 | Parkinson disease  Hoehn & Yahr1.5-2.5 | T: 70.19(9.06)  C: 68.63(10.54) | T: 17/9/8  C: 18/11/7 | Resistance Training  Length of Intervention: 12 weeks  Freq: 3 times a week  Duration: 60 min | CON | TUG |
| Tollár | 2018 | Parkinson disease  Hoehn & Yahr2-3 | T: 67.3(3.4)  C: 67.6(4.1) | T: 35/17/18  C: 20/12/8 | Agility Training  Length of Intervention: 12 weeks  Freq: 3 times a week  Duration: 50 min | CON | TUG |

**Table 4: Details of consistency test for TUG**

| **Side** | **Direct** |  | **Indirect** |  | **Difference** |  |  | **tau** |
| --- | --- | --- | --- | --- | --- | --- | --- | --- |
|  | **Coef.** | **Std. Err.** | **Coef.** | **Std. Err.** | **Coef.** | **Std. Err.** | **P>z** |  |
| AE VS CON | 1.100 | 1.222 | 0.364 | 50.646 | 0.736 | 50.661 | 0.988 | 2.359 |
| AT VS CON | 1.369 | 1.619 | 0.830 | 365.166 | 0.539 | 365.172 | 0.999 | 2.359 |
| BLT VS CON | 2.550 | 1.262 | -5.787 | 7.667 | 8.336 | 7.771 | 0.283 | 2.351 |
| BLT VS RAE | 0.493 | 2.394 | -2.434 | 3.241 | 2.926 | 4.030 | 0.468 | 2.357 |
| COT VS CON | -1.500 | 2.709 | -3.699 | 632.260 | 2.199 | 632.263 | 0.997 | 2.359 |
| CYC VS CON | 0.908 | 1.993 | -1.290 | 447.187 | 2.199 | 447.189 | 0.996 | 2.359 |
| DANCE VS CON | -1.775 | 1.102 | 1.757 | 5.061 | -3.532 | 5.180 | 0.495 | 2.384 |
| EE VS CON | -4.524 | 1.845 | -6.723 | 448.418 | 2.199 | 448.420 | 0.996 | 2.359 |
| ME VS CON | -1.052 | 2.364 | -3.251 | 447.257 | 2.199 | 447.257 | 0.996 | 2.359 |
| PT VS CON | -0.520 | 2.517 | -2.719 | 632.553 | 2.199 | 632.557 | 0.997 | 2.359 |
| QG VS CON | -1.598 | 1.759 | -3.797 | 447.232 | 2.199 | 447.235 | 0.996 | 2.359 |
| RAE VS CON | -4.130 | 1.860 | 5.428 | 4.874 | -9.558 | 5.219 | 0.067 | 2.242 |
| RT VS CON | -0.372 | 1.019 | -6.478 | 4.984 | 6.106 | 5.078 | 0.229 | 2.343 |
| RTI VS CON | 0.300 | 2.544 | -1.623 | 4.931 | 1.923 | 5.519 | 0.727 | 2.398 |
| TAI VS CON | 1.021 | 1.790 | 1.363 | 4.703 | -0.343 | 5.003 | 0.945 | 2.408 |
| TE VS CON | -1.600 | 2.624 | 5.962 | 6.165 | -7.562 | 6.820 | 0.268 | 2.353 |
| VR VS CON | -0.629 | 1.674 | -2.827 | 365.118 | 2.199 | 365.119 | 0.995 | 2.359 |
| WKT VS CON | -0.494 | 0.944 | 2.646 | 6.709 | -3.140 | 6.775 | 0.643 | 2.394 |
| YOGA VS CON | 2.300 | 2.538 | -2.890 | 5.036 | 5.190 | 5.645 | 0.358 | 2.364 |
| DANCE VS RT | -1.429 | 2.566 | 2.009 | 1.642 | -3.439 | 3.048 | 0.259 | 2.351 |
| RT VS RTI | 0.100 | 2.556 | 2.023 | 4.913 | -1.923 | 5.519 | 0.728 | 2.398 |
| RT VS YOGA | 0.700 | 2.596 | 5.890 | 4.946 | -5.190 | 5.645 | 0.358 | 2.364 |
| TAI VS WKT | -1.167 | 2.475 | -1.844 | 2.551 | 0.678 | 3.537 | 0.848 | 2.404 |
| TE VS VR | -1.300 | 2.485 | 6.263 | 6.336 | -7.563 | 6.820 | 0.268 | 2.353 |

**Table 5: Details of consistency test for BBS**

| **Side** | **Direct** |  | **Indirect** |  | **Difference** |  |  | **tau** |
| --- | --- | --- | --- | --- | --- | --- | --- | --- |
|  | **Coef.** | **Std. Err.** | **Coef.** | **Std. Err.** | **Coef.** | **Std. Err.** | **P>z** |  |
| AE VS CON | -1.522 | 1.695 | -0.583 | 60.859 | -0.940 | 60.883 | 0.988 | 3.973 |
| AT VS CON | -1.600 | 4.216 | -1.443 | 632.497 | -0.157 | 632.514 | 1.000 | 3.973 |
| BLT VS CON | -4.771 | 2.250 | 12.441 | 8.816 | -17.212 | 9.099 | 0.059 | 3.715 |
| BLT VS RAE | 5.295 | 3.787 | -4.686 | 4.163 | 9.981 | 5.627 | 0.076 | 3.768 |
| COT VS CON | 0.800 | 4.322 | 3.843 | 632.543 | -3.043 | 632.553 | 0.996 | 3.973 |
| CYC VS CON | -3.600 | 4.055 | 14.126 | 9.166 | -17.726 | 10.064 | 0.078 | 3.820 |
| DANCE VS CON | 4.617 | 2.463 | 12.685 | 9.972 | -8.068 | 10.259 | 0.432 | 4.007 |
| EE VS CON | 3.053 | 2.498 | 6.096 | 365.174 | -3.043 | 365.179 | 0.993 | 3.973 |
| ME VS CON | -0.400 | 3.992 | 2.642 | 632.463 | -3.042 | 632.475 | 0.996 | 3.973 |
| PT VS CON | 3.825 | 2.494 | 13.053 | 9.191 | -9.228 | 9.523 | 0.333 | 3.998 |
| QG VS CON | 2.023 | 2.554 | 13.931 | 9.344 | -11.908 | 9.632 | 0.216 | 3.918 |
| RAE VS CON | -1.417 | 3.008 | 0.958 | 8.344 | -2.375 | 8.873 | 0.789 | 4.083 |
| RT VS CON | 2.811 | 1.946 | 5.855 | 282.894 | -3.043 | 282.898 | 0.991 | 3.973 |
| RTI VS CON | 1.299 | 1.884 | -1.910 | 11.221 | 3.209 | 11.386 | 0.778 | 4.078 |
| TAI VS CON | -2.100 | 4.667 | 10.740 | 9.672 | -12.840 | 10.861 | 0.237 | 3.957 |
| TE VS CON | 0.900 | 3.979 | 18.629 | 9.266 | -17.729 | 10.064 | 0.078 | 3.820 |
| VR VS CON | -1.401 | 4.379 | -3.197 | 4.521 | 1.796 | 6.291 | 0.775 | 4.065 |
| WKT VS CON | -0.420 | 4.385 | -13.262 | 10.060 | 12.842 | 10.862 | 0.237 | 3.957 |
| YOGA VS CON | 1.402 | 4.162 | 3.460 | 4.496 | -2.058 | 6.114 | 0.736 | 4.077 |
| DANCE VS RT | -1.522 | 1.695 | -0.583 | 60.859 | -0.940 | 60.883 | 0.988 | 3.973 |
| RT VS RTI | -1.600 | 4.216 | -1.443 | 632.497 | -0.157 | 632.514 | 1.000 | 3.973 |
| RT VS YOGA | -4.771 | 2.250 | 12.441 | 8.816 | -17.212 | 9.099 | 0.059 | 3.715 |
| TAI VS WKT | 5.295 | 3.787 | -4.686 | 4.163 | 9.981 | 5.627 | 0.076 | 3.768 |
| TE VS VR | 0.800 | 4.322 | 3.843 | 632.543 | -3.043 | 632.553 | 0.996 | 3.973 |

**Table 6: Details of consistency test for Mini-BESTest**

| **Side** | **Direct** |  | **Indirect** |  | **Difference** |  |  | **tau** |
| --- | --- | --- | --- | --- | --- | --- | --- | --- |
|  | **Coef.** | **Std. Err.** | **Coef.** | **Std. Err.** | **Coef.** | **Std. Err.** | **P>z** |  |
| AE VS CON | -4.000 | 2.455 | -0.378 | 65.934 | -3.622 | 65.980 | 0.956 | 1.568 |
| AT VS CON | 0.100 | 1.572 | -8.090 | 632.468 | 8.190 | 632.470 | 0.990 | 1.568 |
| BLT VS CON | -0.978 | 0.573 | -7.015 | 210.725 | 6.037 | 210.726 | 0.977 | 1.568 |
| BLT VS RAE | 3.600 | 1.450 | 8.482 | 2.765 | -4.882 | 3.124 | 0.118 | 1.439 |
| CYC VS CON | -0.800 | 1.902 | 7.190 | 632.870 | -7.990 | 632.871 | 0.990 | 1.568 |
| DANCE VS CON | -0.669 | 1.047 | 7.322 | 364.974 | -7.992 | 364.975 | 0.983 | 1.568 |
| ME VS CON | -2.227 | 2.044 | 5.764 | 447.401 | -7.991 | 447.399 | 0.986 | 1.568 |
| PERT VS CON | -1.000 | 1.870 | 6.990 | 632.770 | -7.990 | 632.771 | 0.990 | 1.568 |
| PT VS CON | 0.230 | 1.749 | 8.220 | 632.279 | -7.990 | 632.281 | 0.990 | 1.568 |
| QG VS CON | 2.170 | 2.217 | 10.160 | 632.122 | -7.990 | 632.122 | 0.990 | 1.568 |
| RAE VS CON | 6.700 | 1.452 | 1.818 | 2.763 | 4.882 | 3.124 | 0.118 | 1.439 |
| RT VS CON | 0.422 | 1.203 | 8.413 | 365.215 | -7.991 | 365.215 | 0.983 | 1.568 |
| WKT VS CON | -0.200 | 2.082 | 7.790 | 631.790 | -7.990 | 631.791 | 0.990 | 1.568 |
| YOGA VS CON | 0.700 | 2.600 | -3.480 | 4.887 | 4.180 | 5.709 | 0.464 | 1.591 |
| RT VS YOGA | -1.320 | 2.290 | 2.861 | 5.332 | -4.181 | 5.709 | 0.464 | 1.591 |

**Table 7: Details of bias test for TUG**

|  | **Coef.** | **Std. Err.** | **z** | **P>|z|** | **[95% Conf. Interval]** |
| --- | --- | --- | --- | --- | --- |
| AT | -.2692352 | 2.030187 | -0.13 | 0.894 | -4.24833 3.709859 |
| BLT | -1.22877 | 1.745137 | -0.70 | 0.481 | -4.649175 2.191635 |
| CON | 1.099502 | 1.221509 | 0.90 | 0.368 | -1.294612 3.493615 |
| COT | -.4004989 | 2.971489 | -0.13 | 0.893 | -6.224511 5.423513 |
| CYC | 2.007669 | 2.336283 | 0.86 | 0.390 | -2.571362 6.586701 |
| DANCE | -.5162131 | 1.62131 | -0.32 | 0.750 | -3.693923 2.661497 |
| EE | -3.424901 | 2.212039 | -1.55 | 0.122 | -7.760418 .9106152 |
| ME | .0474907 | 2.661298 | 0.02 | 0.986 | -5.168557 5.263539 |
| PT | .5794968 | 2.797783 | 0.21 | 0.836 | -4.904058 6.063052 |
| QG | -.4985414 | 2.142472 | -0.23 | 0.816 | -4.697709 3.700626 |
| RAE | -1.776594 | 2.177578 | -0.82 | 0.415 | -6.044568 2.491381 |
| RT | .4937857 | 1.582124 | 0.31 | 0.755 | -2.60712 3.594692 |
| RTI | 1.000652 | 2.551414 | 0.39 | 0.695 | -4.000027 6.00133 |
| TAI | 2.160969 | 2.054206 | 1.05 | 0.293 | -1.8652 6.187139 |
| TE | .750521 | 2.670334 | 0.28 | 0.779 | -4.483237 5.984279 |
| VR | .4707445 | 2.071578 | 0.23 | 0.820 | -3.589474 4.530963 |
| WKT | .6660878 | 1.530522 | 0.44 | 0.663 | -2.333681 3.665856 |
| YOGA | 2.345408 | 2.569032 | 0.91 | 0.361 | -2.689802 7.380618 |

**Table 8: Details of bias test for BBS**

|  | **Coef.** | **Std. Err.** | **z** | **P>|z|** | **[95% Conf. Interval]** |
| --- | --- | --- | --- | --- | --- |
| AT | .0778699 | 4.544225 | 0.02 | 0.986 | -8.828648 8.984388 |
| BLT | 2.217418 | 2.871437 | 0.77 | 0.440 | -3.410495 7.845331 |
| CON | -1.522136 | 1.695213 | 0.90 | 0.369 | -4.844692 1.800421 |
| COT | -.7221329 | 4.642667 | -0.16 | 0.876 | -9.821593 8.377327 |
| CYC | -2.144431 | 4.182706 | -0.51 | 0.608 | -10.34238 6.053522 |
| DANCE | 3.543533 | 2.916881 | 1.21 | 0.224 | -2.173448 9.260515 |
| EE | 1.531116 | 3.014845 | 0.51 | 0.612 | -4.377871 7.440103 |
| ME | -1.922095 | 4.337485 | -0.44 | 0.658 | -10.42341 6.57922 |
| PT | 2.937356 | 2.936063 | 1.00 | 0.317 | -2.817222 8.691934 |
| QG | 1.276718 | 3.021902 | 0.42 | 0.673 | -4.646102 7.199538 |
| RAE | -2.664601 | 3.237945 | -0.82 | 0.411 | -9.010857 3.681655 |
| RT | 1.289131 | 2.582098 | 0.50 | 0.618 | -3.771688 6.349951 |
| RTI | -.3149531 | 2.480622 | -0.13 | 0.899 | -5.176882 4.546976 |
| TAI | -1.106417 | 4.498851 | -0.25 | 0.806 | -9.924004 7.71117 |

**Table 9: Details of bias test for Mini-BESTest**

|  | **Coef.** | **Std. Err.** | **z** | **P>|z|** | **[95% Conf. Interval]** |
| --- | --- | --- | --- | --- | --- |
| AT | -4.097556 | 2.913914 | -1.41 | 0.160 | -9.808722 1.61361 |
| BLT | -3.019567 | 2.51966 | -1.20 | 0.231 | -7.958011 1.918877 |
| CON | -3.997695 | 2.453761 | -1.63 | 0.103 | -8.806978 .8115884 |
| CYC | -4.797686 | 3.104802 | -1.55 | 0.122 | -10.88299 1.287614 |
| DANCE | -4.667032 | 2.667735 | -1.75 | 0.080 | -9.895695 .5616321 |
| ME | -6.224506 | 3.193829 | -1.95 | 0.051 | -12.4843 .0352849 |
| PERT | -4.997686 | 3.084836 | -1.62 | 0.105 | -11.04385 1.048481 |
| PT | -3.767684 | 3.01343 | -1.25 | 0.211 | -9.673897 2.13853 |
| QG | -1.827691 | 3.306873 | -0.55 | 0.580 | -8.309044 4.653661 |
| RAE | 1.639173 | 2.823653 | 0.58 | 0.562 | -3.895086 7.173432 |
| RT | -3.575866 | 2.732982 | -1.31 | 0.191 | -8.932412 1.78068 |
| WKT | -4.197687 | 3.218139 | -1.30 | 0.192 | -10.50512 2.10975 |
| YOGA | -4.293688 | 3.299626 | -1.30 | 0.193 | -10.76084 2.173461 |
